# ‘It’s going to change the way we train’: Qualitative evaluation of a transformative faculty development workshop

**DOI:** 10.1007/s40037-021-00687-4

**Published:** 2021-10-25

**Authors:** Caroline Choo Phaik Ong, Yang Yann Foo, Fong Yee Chiu, Debra Nestel

**Affiliations:** 1 KK Women’s and Children’s Hospital, Singapore Health Services (SingHealth), Singapore, Singapore; 2 SingHealth Duke-NUS Academic Medical Centre, Singapore, Singapore; 3 Academic Medicine Education Institute, Office of Education, Duke-NUS Medical School, Singapore, Singapore; 4 Faculty of Medicine, Nursing & Health Science, Monash University, Melbourne, Australia; 5 Department of Surgery (Austin), University of Melbourne, Melbourne, Australia

**Keywords:** Transformative learning, Educator identity formation, Faculty development program, Long-term program evaluation

## Abstract

**Introduction:**

Relatively little is known about faculty development (FD) activities that help participants achieve sustainable behavioral change. This qualitative study evaluated the medium- to long-term impact of a FD workshop informed by transformative learning (TL) theory. It aimed to discover which aspects of FD prompted healthcare professionals (HPs) to adopt effective teaching and learning practices.

**Methods:**

Seventeen participants were interviewed between January and July 2020, 7 to 30 months after the workshop. Purposeful sampling strategies were used to collect data and analysis was performed using reflexive thematic analysis.

**Results:**

Four themes were identified: perspectival shift in educational practice, re-affirmation of current practices, becoming an educator, and valuing FD that accommodates HPs’ multiple communities of practice (CoPs). Workshop activities foregrounding critical discourse and reflection helped participants gain new knowledge and deeper understanding of education. TL was likely when participants already identified as an educator in addition to their HP identity. Additionally, a workplace CoP determined the type and level of support affecting HPs’ development as educators.

**Discussion:**

Aspects of FD that prompted HPs to adopt effective teaching and learning practices included initiatives that catalyzed critical discourse and reflection. Readiness for TL is promoted when HPs have a strong educator identity because of workplace educator CoPs. Future research could explore effecting sustainable post-workshop behavioral change in HPs through the strengthening of workplace educator CoPs. To do this, institutions could send co-located HPs from different disciplines to the same FD program.

**Supplementary Information:**

The online version of this article (10.1007/s40037-021-00687-4) contains supplementary material, which is available to authorized users.

## Introduction

Health professionals (HPs) often have inadequate teaching skills to facilitate clinical learning in the workplace [1]. Busy HPs prefer short-duration faculty development (FD) workshops that achieve outcomes mainly at levels 1 (Reaction) and 2 (Learning) of Kirkpatrick’s evaluation framework [2], while there is a gap in the literature regarding how to help participants achieve sustainable behavioral change [3, 4]. One approach could be through evidence-informed intentionally designed FD [2].

Transformative learning (TL) is a widely appreciated meta-theory described as *“learning that challenges established perspectives, leading to new ways of being in the world”* [5]. Mezirow first described TL as a multi-phase model [6]: perspective transformation begins when individuals experience a disorienting dilemma (something that seriously challenges their established perspectives). They become dissatisfied with these perspectives, start to critically reflect on their underlying assumptions and, through engaging in critical discourse with others, they plan and implement actions to acquire new perspectives [6]. The model has undergone several revisions [7, 8] with some iterations asserting that the phases are not necessarily sequential nor all essential [8]. Mezirow [9] identified critical reflection and critical discourse as major elements of perspective transformation. Critical reflection requires individuals to recognize assumptions they have uncritically assimilated, so as to make judgements based on rational evaluation of their beliefs and values [10]. It may occur independently or through engaging others in critical discourse where all discussants share their views freely [10].

Besides Mezirow’s TL (psychocritical version), other types of TL have been propounded [8] that include psychoanalytic TL, a process of individuation involving deeper understanding of one’s inner self and self-responsibility to discover new talents [11]. In this study, we combine psychocritical and psychoanalytical TL approaches, where the emphasis is on the individual.

### Transformative learning (TL) theory for faculty development (FD)

At SingHealth, we designed a full-day FD workshop for HPs responsible for clinical procedural skills training for pre- and post-registration HP students and trainees. We chose TL [6, 9, 12, 13] to inform workshop design for TL’s potential to effect enduring changes to individuals’ behavior [14, 15], in domains of general [13] and HP education [5]. The workshop design utilized strategies to foster TL [1, 5, 16, 17] by providing learning experiences that stimulate critical reflection, help participants formulate action plans, and link theory with practice (see Appendix 1 in the Electronic Supplementary Material [ESM] for workshop strategies following TL principles).

This qualitative study sought to evaluate a TL-informed FD workshop 6 to 36 months after completion. Using TL as a theoretical framework [18, 19], our study aimed to discover which aspects of formal FD prompt HPs to adopt effective teaching and learning practices.

## Methods

“Optimizing Learning with Task Trainers” is a 7-hour single day FD workshop comprising multiple short didactics interleaved with discussions and simulated teaching activities (see Appendix 2 in ESM for workshop details). Four workshops took place between 2017 and 2019, each with 21–24 attendees. The workshop attendees were HPs (doctors, nurses and allied health professionals [AHPs]) from different specialties, working in acute hospital and community care, responsible for teaching procedural skills that ranged from basic procedures to complex multistep surgeries. All taught learners in the workplace; some also organized task trainer-based workshops. Our study received institutional ethical board exemption (CIRB Ref No: 2019/2909).

### Data collection

CFY and FYY recruited by inviting all local workshop participants via email. CFY took consent and arranged interviews. FYY conducted the semi-structured interviews, assisted by CFY. Audio-recorded interviews were transcribed verbatim, de-identified (CFY) and checked for accuracy (FYY).

We used two purposeful sampling methods. First, to establish broad understanding of whether participants benefited from workshops and how that might change their teaching practice (or otherwise), we applied the maximum variation sampling technique [20]; we ensured that our interviewees included individuals from different health professions, every workshop cohort, with differing levels of teaching experience. Our preliminary analysis showed that participants who saw themselves as part of a teaching community, used what they learnt in the workshop to effect change in their teaching practice. Second, with this finding, we adopted analytically focused sampling [20] to recruit more participants from the same health profession (Participants P14, P15 and P17) who spoke about effecting change, because they belonged to a particular health profession educator community. Data sufficiency was reached after 17 interviews.

We collected data between January and July 2020. Due to COVID-19, Zoom videoconferencing replaced face-to-face interaction for 14 of 17 interviews. Of 63 participants invited, 22 (35%) agreed to be interviewed.

Semi-structured interviews were based on a topic guide (ESM, Appendix 3) with questions exploring the participants’ workshop, what they could recall and how it changed their teaching practice. Aiming to understand the translation gap between knowledge provision and practice change, we asked additional questions encouraging interviewees to freely examine whether and how they were (un)able to apply workshop-learning in their workplace. The fifth interview onwards included an *aide-mémoire* to enhance participant workshop recall—a PowerPoint summary of workshop topics and photographs (ESM, Appendix 4). During iterative data collection and analysis, we modified the topic guide from the sixth to eleventh interviews to critically review themes.

### Data analysis

Data were analyzed using reflexive thematic analysis (TA) [21, 22], a method for identifying and interpreting patterns of shared meaning across datasets, united by a central concept [23, 24]. The analysis was conducted from a critical realist position which asserts that *“reality is ‘out there’ but access to it is always mediated by sociocultural meanings”* [22, p. 21], such as *“culture, language, and political interests rooted in factors such as race, gender, or social class”* [25]. Even though our study was underpinned by Mezirow’s TL theory [6, 12, 13], as congruent with reflexive TA [23], we did not only generate themes deductively by imposing TL concepts on our interpretation of the data, we also inductively analyzed the dataset which spoke to concepts beyond TL; specifically, concepts related to communities of practice (CoP) [26] and workplace CoP [27].

CO, DN and FYY read the first four transcripts and made familiarization notes regarding interesting aspects of the data relevant to our research questions. The remaining 13 transcripts were divided equally amongst coders with one extra to CO. We each identified broad patterns within the transcripts, and at regular meetings discussed the codes constructed. We then developed candidate themes based on the entire dataset. To ensure dependable findings, we re-examined the data using insights gained during analysis. During review, we realized that one theme (“Perspectival shift in educational practice”) contained more than one organizing concept, so we developed the main concept (“the significance of workshop validating participants’ prior experience as educators”) as another theme (“Deepening, validating and re-affirming current practices”). In the final stages, all authors reviewed, defined and named the themes before choosing data excerpts that best represented each theme for a more in-depth analysis.

We were reflexive throughout and constantly questioned assumptions that could shape analysis. To resolve disagreements in interpretation we discussed issues until we achieved consensus. To ensure confirmability, CFY kept an audit trail and meticulously documented the rationale behind decision-making.

The research team comprised a surgeon educator from Singhealth (CO), an experienced qualitative researcher from a university linked to SingHealth (FYY), the study grant administrator (CFY) and an experienced HP educator and qualitative researcher from a separate institution (DN). Only CO had working relationships with several study participants due to her clinical education leadership role. DN and CO designed and taught the FD workshop. To mitigate possible influence, DN and CO were not involved in recruitment and interviews, and remained unaware of the participants’ identities apart from their HP role, due to the sampling strategy.

## Results

The 17 study participants (11 [65%] doctors, 5 [29%] AHPs, 1 [6%] nurse) were representative of local workshop attendees (43 [68%] doctors, 14 [22%] AHPs, 6 [10%] nurses). Participants were interviewed between 7 and 30 months after the workshop (*m* = 18). Interviews ranged from 26–70 minutes (*m* = 44).

All participants demonstrated knowledge gains as they could describe some content taught at the workshop, while 12 participants described how they had implemented teaching skills acquired from the workshop. Most participants described how perspective transformation sparked by the workshop was influenced by their teaching experiences, workplace roles and support (Appendix 5 [ESM] and Tab. 1). Appendix 5 (ESM) is a table summarizing the study’s themes with representative quotes; Tab. 1 shows how the study themes align with TL.

**Table 1:** Correlation of study findings with Transformative Learning (TL) theory

**TL phases** [7] 1. A disorienting dilemma 2. A self-examination with feelings of guilt or shame 3. A critical assessment of epistemic, sociocultural or psychic assumptions 4. Recognition that one’s discontent and the process of transformation are shared and that others have negotiated a similar change 5. Exploration of options for new roles, relationships and actions 6. Planning of a course of action, renegotiating relationships and negotiating new relationships 7. Acquisition of knowledge and skills for implementing one’s plans 8. Provisional trying of new roles 9. Building of competence and self-confidence in new roles and relationship 10. A reintegration into one’s life on the basis of conditions dictated by one’s perspective		**THEME 1**: **Perspectival shift in educational practice** *A substantive shift in concepts of learning and/or teaching based on experiences in the workshop and subsequent application in practice*
		**1.1** New ideas, practices or approaches **1.2** New insights into existing concepts about education **1.3** New relationships foster critical discourse for learning
		**THEME 2**: **Deepening, validating, and re-affirming current practices** *The workshop experience serves to clarify and structure prior ideas on teaching–learning and to affirm the associated beliefs and values*
		**THEME 3**: **Being and/or becoming an educator** *Health professionals (HPs) identify as clinicians and not all HPs have developed an educator identity in addition to the HP identity. Attunement and growth mindset is necessary for transformative learning*
		**3.1** Having or developing an educator identity in addition to the HP identity **3.2** Attunement and growth mindset are necessary for transformative learning
		**THEME 4**: **Valuing faculty development that accommodates HPs’ multiple communities of practice** *Faculty development for HPs should recognize competing clinical priorities and the workplace community of practice*
		**4.1** Design of faculty development sensitive to HPs’ complex clinical/workplace needs **4.2** The workplace educator community of practice influences transformative learning gained through formal faculty development
**Phase**	**Study findings that correlate with TL**	
** 1–3**	THEME 1 (Subthemes 1.1 and 1.2)	
	*Confirmatory finding:* A review of empirical evidence for Mezirow’s model found that the 10 phases are not necessarily sequential nor universally present and broadened the original definition of disorientating dilemma triggered by acute personal crisis [6] to include disequilibrium provoked by experiences incongruent with established meaning perspectives [37]. Our study findings are consistent with this. *Contribution to TL:* Several of our participants describe disorienting dilemma in terms of positive rather than negative emotions (e.g., excitement at new conceptual understanding). Many of the original TL studies involved participants with illness or from marginalized groups [37], while a recent education study described emotions ranging from joyful to unpleasant [38]. We suggest that strong emotion is required as catalyst while the type of emotion is less relevant	
	THEME 2	
	*Confirmatory finding: *Self-examination leads to critical assessment of epistemic assumptions about teaching–learning. A deeper understanding reinforces and improves on previous practices, by emphasizing incompletely recognized aspects of practice	
** 4**	THEME 1 (Subtheme 1.3)	
	*Confirmatory finding:* Reflection and critical discourse is important for TL [13]. The workshop provided a new opportunity to discuss education with educators that allowed a deeper understanding of concepts and stimulated ideas	
** 5–9**	THEME 1 (Subthemes 1.1 and 1.2)	
	*Confirmatory finding:* The workshop utilized strategies to foster TL (ESM, Appendix 1) [1, 5, 16, 17]. Additional TL research highlights the influence of context, the importance of relationships and other factors like feelings and support structures that promote and inhibit perspective transformation [17]. Our study findings confirm the efficacy of such strategies in promoting TL and reinforce the influence of contextual support factors	
**10**	THEME 3 (Subthemes 3.1 and 3.2); THEME 4 (Subtheme 4.2)	
	*Confirmatory findings*: 12/17 participants described enactment of TL. They were those with stronger educator identity, displayed educator characteristics of reflecting on teaching practice, valued FD and regularly engaged with others on education matters within their workplace communities of practice. These findings suggest that a strong educator identity with a supportive “pro-education” community of practice promotes enactment of TL. Our study corroborates Mezirow’s revised TL [12] where he placed more emphasis on the social/relationship aspects of perspective transformation	

### Theme 1: Perspectival shift in educational practice

Participants reported a substantive shift in their conceptualization of learning and/or teaching based on their workshop experience and subsequent application in practice. “*Because it’s going to change the way we train … that’s why it’s a great impact”* (P14, AHP). Three participants used similar words to describe the workshop: *“opened my eyes”* (P1, doctor), *“eye-opening”* (P3, doctor) and *“an eye-opener”* (P17, AHP).

#### New ideas, practices or approaches

Some participants pointed to specific ideas encountered in the workshop that profoundly changed how they conceived learning and/or teaching. Other participants pointed to new resources and/or tools that helped them make concrete changes to their teaching practice to align with new understandings.

#### New insights into existing concepts about education

Some participants, when prompted, could identify teaching methods used in the workshop that modelled educational concepts taught in the workshop and the underpinning educational theories. *“So I remember … the facilitators themselves are applying the concepts throughout the whole workshop … we find that very interesting”* (P15, AHP). Their experiences as learners reinforced this—for example, there was a realization that course planning was important because they were required to experience the plan in the workshop. For others, being presented with a formal educational framework helped them appreciate the educational concept more holistically. That is, it offered a rationale for teaching practice.

#### New relationships foster critical discourse for learning

The workshop allowed new introductions. It also created opportunity for critical discourse between some who were familiar workplace colleagues but had previously never engaged in education-related discussion. This was valued by those without regular access to like-minded educators.* “In my department … there are only two other people who have real interest … many people would say ‘yeah I’m interested in teaching’ but if you tell them about Peyton model or Kolb model or learning models … it’s not something they are comfortable with”* (P7, doctor).

### Theme 2: Deepening, validating, and re-affirming current practices

Some participants had previously encountered the concepts taught in the workshop through attending other formal FD, or the presented concepts were consistent with their teaching experiences. Their workshop experience affirmed their beliefs and values about learning and/or teaching. For others, the workshop helped to clarify and structure their approach to learning and/or teaching.

### Theme 3: Being and/or becoming an educator

#### Having or developing an educator identity in addition to the HP identity

Most HPs have a primary professional identity as doctor, nurse or AHP. Generally, all HPs teach as part of their role. Increased involvement in education may be associated with formal appointment as HP educator or driven by one’s own interest in teaching. Only some have developed an educator identity in addition to the HP identity. *“This is just something I have been having an interest in … I always consider myself an amateur educator”* (P11, doctor).

Experience as HP is not always correlated with experience as educator. Some junior HPs have taught much more than senior HPs. However, it is a common belief among participant HPs that senior HPs with more clinical experience should be “better” teachers. Some HPs have been teaching for many years, yet do not recognize that an educator requires professional development in knowledge, skills and attitudes different from clinical training. They may call themselves HP educators because of formal appointment yet do not demonstrate educator attributes of humility, willingness to self-reflect and learn about education matters. *“So I’m at the stage where … there’s an SOP [standard operating procedure]. Everything has sequence, steps, and I don’t try to vary. By the time you have to vary, it’s not meant to be teaching”* (P7, doctor).

Depending on their clinical discipline, many HP educators do not routinely receive formal FD and some feel inadequately prepared for the role. Formal appointment and/or formal FD helps to reassure HP educators that they are doing the right thing.

#### Attunement and growth mindset are necessary for TL

Whether the HP has a strong formalized educator identity or not, the passionate educator demonstrates regular self-reflection on teaching practice, seeks out opportunities to try new teaching–learning methods, solicits feedback and discusses education with others. Several participants described enactment of TL arising directly or indirectly from the workshop. The pandemic cancelled many teaching activities, which prevented a few from implementing planned changes in teaching practice. Nevertheless, keen educators used creative alternatives to test workshop-acquired skills. For example, P2 tried out feedback techniques during clinical duties (teaching caregivers of her pediatric patients), while P8 practiced teaching her son how to fry an egg.

### Theme 4: Valuing FD that accommodates HPs’ multiple communities of practice (CoP) [26]

#### Design of FD sensitive to HPs’ complex clinical/workplace needs

Due to competing clinical priorities, HPs value focused, relevant formal FD with regular reinforcement by structured refresher activities. Our interviewees vividly remembered workshop activities of simulated teaching, role-play, games and photo-taking. They appreciated the interaction with fellow educators, both familiar and new acquaintances, and prompts to reflect on their teaching–learning practices. *“It was very engaging in the sense that we had all the group activities, then there was the bonding part as well … colleagues from [the same institution]. So we had a good time.”* (P12, doctor).

Some interviewees lamented forgetting some workshop-learning because of lack of practice due to irregular teaching opportunities and competing clinical commitments. All participants had received a feedback survey two months after the workshop as a prompt for reflection. Nevertheless, at the time of our study interview 7–30 months later, many had forgotten details of educational concepts taught in the workshop. Several suggested having more follow-up reminders at regular intervals.

#### The workplace educator CoP influences TL gained through formal FD

HPs engage in education matters opportunistically, mostly with clinical colleagues in their workplace CoP. The educator expertise available within the workplace CoP varies between departments. The departmental culture also affects the value placed on education. Both factors affect the support and mentorship given to HP educators as well as the critical discourse available.

## Discussion

We found that the workshop achieved its goal of providing knowledge with perspective transformation, but complex factors, beyond the effect on individuals, affected modification of teaching practices. During inductive analysis, we found practice change was influenced by additional workplace factors, which is congruent with FD literature highlighting the importance of social learning and context [27–29].

The workshop designed following TL principles (ESM, Appendix 1) had some strategies that worked well: the educational theory-based content was novel to many, which helped trigger TL; the workshop format prioritized reflection and critical discourse to reinforce learning; and simulated teaching activities built confidence and competence in new teaching skills. Other workshop strategies of a written action plan and post-workshop follow-up were less effective in promoting complete enactment of TL (Mezirow’s phase 10). Further, most participants shared with us that insights sparked by their workshop experience were developed through subsequent reflection, discussion and testing of new ideas. Like higher education teachers [30], they appreciated “explicit guidance tools” [30] to help implement change in teaching practice, such as feedback models and lesson plan templates from the workshop.

A few participants did not report any practice of workshop-acquired skills, that is, they described some perspective transformation (Mezirow’s earlier phases) but no enactment of TL. Our analysis (Tab. 1) suggests that two factors greatly influence whether HPs will adopt new education practices: the type of educator identity and the type of workplace CoP [26] to which they belong. Specifically, HPs who teach have variable conceptions of teacher identity ranging from ‘hierarchical’ (secondary to their clinical/researcher roles) to ‘compartmentalized’, ‘intersecting’ or ‘merged’ [31]—with the latter two groups more likely to value teaching and professional development as teachers [31, 32]. Our study viewed teacher identify formation with an individualist orientation that highlighted the tensions faced by our participants in identity juggling [32]. We found that the participants that enacted change in teaching practice following the workshop were those with strong(er) educator identity, attuned to critically reflect on teaching practice [31]. They readily reflected about their limitations as educators and were open to questioning assumptions underlying their practices and trying new teaching methods [13].

Unlike studies on junior teachers where increased teaching experience [33] and/or cognitive maturity [17, 34] improved critical reflection, we found that HPs’ propensity to reflect on teaching practice was unrelated to their teaching or clinical experience. We suspect that habitual reflexive practice is an attribute related to educator identity development that is augmented by FD. Although experienced HPs commonly reflect on clinical practice, HPs without a developed educator identity may not be accustomed to reflecting on teaching practice, since “invisible” teacher identity (teacher identity subsumed within clinician identity) is associated with low motivation to improve teaching ability [32].

The social-relational perspective on clinician teacher identity formation [29] highlights how the tensions between the HP and educator identity reported by our participants affect their perception of legitimacy and social value as teachers; this, in turn, influences motivation to [32] engage with FD. TL has been shown to influence formation of professional identity in HP students [5] by making sense of experiences [35]. We suggest that transformative FD activities help HPs develop and reinforce their educator identity, negotiated through socialization [31, 36]. In turn, HPs with a strong educator identity with attunement, when attending formal FD are “primed” for TL. Together with workplace support, FD that is sensitive to teacher identity will encourage HP educators who enjoy teaching and seek improvement as teachers [31].

In our study, HPs with a strong educator identity tended to belong to a “pro-education” CoP [29]. Their CoPs afforded them mentorship guidance, regular education discussions with interested colleagues and protected time from clinical duties to pursue education activities. The important contribution of CoP to FD is highlighted by O’Sullivan and Irby who recommend a dual CoP model: one CoP within the FD program and one CoP within the workplace [27]. In our context, HPs interact mostly with co-located colleagues at the workplace, where education discourse is limited to a few like-minded colleagues. Hence, our HPs conceptualize the CoP of teaching practice at the workplace differently as a small educator workplace CoP nested within the larger clinical workplace CoP.

Recognizing these different types of CoPs will improve design of FD for HPs. A systematic review of teaching effectiveness of FD initiatives found that longitudinal FD programs yield more sustainable outcomes over short programs [2]. However, standalone workshops can bring about profound perspective transformation if designed with CoP and TL considerations. Beyond creating a transitory within-workshop CoP of educators, the workshop should aim to develop the workplace educator CoP by inspiring participants to “bring back” TL with them to their workplaces: by modelling their changed teaching practices and discussing their new learning with colleagues. At the systems level, institutions should take a broader view of staff professional development [27]. At present, FD is typically planned for individual HPs based on their teaching role. With thoughtful consideration of the institution’s existing networks of workplace and educator CoPs, the impact of FD for a few individuals may be amplified by propagation to others; for example, a natural way to expand the workplace educator CoP is to send a few co-located HPs from different disciplines to the same FD program. A related FD finding was the participants’ expressed need for “refresher” FD as longitudinal support. Planning structured FD for prolonged engagement is a form of intentional community building that boosts FD approaches [2].

### Strengths and limitations of study

Our study is one of few HP education studies investigating TL in different health professions and graduate learners [5]. A feature of our study is data collection timing extended to track longer-term changes beyond immediate post-workshop experience. Our study demonstrated workshop outcomes at Kirkpatrick Level 3 (Behavior). One study limitation is the fact that it was conducted in a single institution, albeit large, comprising 4 acute hospitals, 5 national specialty centres, 2 community hospitals and several primary care clinics. Additionally, qualitative research methodology has been criticized for capturing a “retrospective snapshot” of the learning experience and risks confusing TL with learning arising from natural development [17, 37]. We countered memory decay by using an aide-mémoire [17] and triangulated participant descriptions of TL enactment with other sources of information such as institution reports of new teaching programs created. We possibly recruited proportionately more study participants with TL because the same educator characteristics that prime for TL increased enthusiasm for participating in education research.

## Supplementary Information

Appendix 1: Key aspects of workshop design following Transformative Learning TL principles

Appendix 2: Instructional design of the Optimizing Learning with Task Trainers OLTT workshop reported following Extension to the CONSORT and STROBE statements guidelines

Appendix 3: Topic guide

Appendix 4: Aide- Memoire: Powerpoint slide summary of key workshop topics and photographs to trigger participant recall during the interview

Appendix 5: Summary table of study themes and subthemes with representative quotes
