# Supplementary material for: ‘It’s going to change the way we train’: Qualitative evaluation of a transformative faculty development workshop
Source: Perspect Med Educ. 2021 Oct 25;11(2):86–92. doi: 10.1007/s40037-021-00687-4 (PMC8543424; doi:10.1007/s40037-021-00687-4)
Supplement: Supplementary file 1 — Appendix 1: Key aspects of workshop design following Transformative Learning TL principles [file 40037_2021_687_MOESM1_ESM.pdf]

Appendix 1: Key aspects of workshop design following Transformative Learning TL principles.

**Mezirow's Ten Phases of TL learning** [1,2]	**Workshop strategies** (See Appendix 2 for workshop details)	**Comments on efficacy of strategies based on study findings**
1. A disorienting dilemma<br>2. A self-examination with feelings of guilt or shame<br>3. A critical assessment of epistemic, sociocultural, or psychic assumptions	Introduces education theories and frameworks of skills development, both as prereading and through short didactics during workshop. Participants are invited to examine each concept using prior frame of reference, by applying it to a familiar teaching-learning experience	There were different triggers for disorientating dilemma: often it was one or more of the new educational theories or skills learnt; for a few participants, it was the fundamental concept of teaching-learning underpinned by theory-based structure. We could not predict the type of trigger based on participant's level of seniority nor teaching experience. The workshop content which aimed to provide a broad but superficial overview, appeared to trigger perspective transformation in most of the participants.
4. Recognition that one's discontent and the process of transformation are shared and that others have negotiated a similar change<br>5. Exploration of options for new roles, relationships, and actions	Throughout workshop, personal reflection is encouraged with planned time for individual written exercises.<br>For each concept introduced, time is allocated for peer and /or small-group discussions. Discussion groupings are actively re-mixed, to expose everyone to a variety of different experiences and ideas.	Majority of participants did not recollect written individual reflection, but several appreciated that the workshop allowed them space and time for reflection on teaching practice.<br>Discussion within the workshop was particularly memorable since every participant highlighted that this aspect contributed greatly to their learning from the workshop.
6. Planning of a course of action, **renegotiating relationships and negotiating new relationships**<br>Phase 6 amended in 1991 with addition of words in *italic*[1,3]	Half of workshop time involves creating a learning plan and implementing it in a simulation activity.<br>Group discussions are prioritised to encourage formation of new relationships with explicit invitation to develop new collaborations after the workshop.<br>Participants write individual action plan for implementing change in teaching activities after the workshop (see below)	The learning plan simulation activity within the workshop, worked better for those who came to the workshop with specific projects in mind; in particular, the programmes had better chance of successful implementation when members of the same collaborative group attended the workshop together.<br>After the workshop, the within-workshop relationships did not persist for those without prior relationships. However, a few participants maintained relationship with the main workshop faculty (CO) who mentored their implementation of new teaching programmes.
7. Acquisition of knowledge and skills for implementing one's plans	Workshop didactics and simulated teaching activities	Strategies were effective as evidenced by study findings (see Table 1)
8. Provisional trying of new roles<br>9. Building of competence and self-confidence in new roles and relationship	Simulated teaching activities	
10. A reintegration into one's life on the basis of conditions dictated by one's perspective	Participants write individual action plan at workshop for post workshop implementation. A post-workshop feedback survey is sent 2 months after the workshop as prompt for reflection and encouragement to implement action plan.	The feedback survey was too soon (at 2 months) for most participants to implement change while most had implemented some changes by the time of the study (at 7-30 months). The qualitative interview for the study also served as an active intervention to enhance reflection and workshop learning. Based on our study's findings, we now include an aide-memoir with additional questions as workshop follow-up that aims to substitute the reflection prompt of the qualitative interview.

REFERENCES

1. Kitchenham A. The Evolution of John Mezirow's Transformative Learning Theory. JTED. 2008;6:104-23.

2. Mezirow J. Perspective Transformation. Adult Educ. 1978;28.

3. Mezirow J. Transformative Dimensions of Adult Learning. Jossey-Bass; 1991.
