# Supplementary material for: ‘It’s going to change the way we train’: Qualitative evaluation of a transformative faculty development workshop
Source: Perspect Med Educ. 2021 Oct 25;11(2):86–92. doi: 10.1007/s40037-021-00687-4 (PMC8543424; doi:10.1007/s40037-021-00687-4)
Supplement: Supplementary file 2 — Appendix 2: Instructional design of the Optimizing Learning with Task Trainers OLTT workshop reported following Extension to the CONSORT and STROBE statements guidelines [file 40037_2021_687_MOESM2_ESM.pdf]

Appendix 2: Instructional design of the Optimizing Learning with Task Trainers OLTT workshop reported following Extension to the CONSORT and STROBE statements guidelines[1]

CONTEXT

Procedural skills form an integral part of clinical patient care provided by all types of health professionals (HPs) including doctors, nurses and allied health practitioners. The study institution comprises several acute hospitals, step-down care facilities and general clinics that partner with various educational institutions to provide clinical training for pre- and post-registration HP students and trainees. Procedural skills training is commonly done by supervised workplace learning supplemented with simulation-based education. HP educators leading skills development in the institution receive variable amounts of formal faculty development. Prior to this workshop, there was no local programme designed specifically for clinical procedural skills educators.

WORKSHOP DESIGN AND OVERVIEW

Two key constraints of workshop design were the limited time of busy HP educators and a heterogeneous audience with different clinical and educator experiences. The OLTT full-day workshop is delivered yearly as an isolated faculty development course. The workshop content covers basics of simulation-based education curriculum design including feedback together with educational theories and frameworks specific to skills development. The workshop structure uses strategies to foster transformative learning[2, 3] with simulated teaching activities, individual written exercises and peer discussion to facilitate reflection and the experience of implementing a learning plan created with guidance. Participants receive a follow-up email survey 2 months after the workshop, with questions to prompt reflection and commitment to practice change.

COURSE OBJECTIVES

This workshop aims to support participants in gaining understanding of fundamental educational processes for skills development using task trainers.

SPECIFIC LEARNING OBJECTIVES

After the workshop, participants will be able to:

1. Discuss benefits of using task trainers in skills development
2. Discuss limitations of using task trainers in skills development
3. Describe models of skills development and training
4. Describe the role of feedback in task trainer-based skills development
5. Design a learning plan that uses a task trainer to support the development of a skill

PRECOURSE PREPARATION

The course organizers arrange task-trainers and group activities appropriate for the participants' health profession and teaching experience. One week before the workshop, participants are emailed the pre-course readings of 3 journal articles and are advised to read at least one in preparation.

WORKSHOP SETTING

The participants are seated 3-to-a-table in separate table groups yet close enough for whole class discussion. Faculty-learner ratio is maintained at a minimum of 1:8 to allow facilitation. Throughout the workshop, activities require re-grouping and individual movement, to encourage opportunities for participants to make new connections.

WORKSHOP STRUCTURE

Full-day workshop (7 hours) starting with registration and breakfast, followed by the workshop that incorporates one morning tea-break and one lunch-break.

**Section**	**Program aims**	**Activity description**
Introduction and Orientation to workshop	Setting the expectations for social learning and understanding participants' frames of reference[4]	Faculty and participant introduction followed by orientation to workshop structure and materials. Participants are invited to use the course worksheet regularly for individual written reflections to enhance learning.
Simulation fundamentals	Discussion on pre-course readings	Facilitated peer and small group sharing on learning points from pre-course readings.
	Task-trainers	Short didactic followed by individual written reflections and table discussions.
Skills development	Major theories of skills development: Fitts and Posner[5] Kolb[6], Peyton[7], Mastery learning[8], Sawyer[9]	The theories are introduced using a mixture of didactics interspersed with individual written reflections, table and whole class discussions. Participants are invited to contextualize the theories to personal experience.
		Simulated teaching activity using Peyton's model: on each table, one participant teaches a simple skill using a familiar task trainer (suturing, venipuncture or ultrasound probe positioning) to a fresh learner (first year medical student volunteer) while the table partners act as observers. This is followed by table debriefing that includes the student learner's feedback on his/her experience.

Role of feedback	Generic and specific skills-teaching feedback models:<br>DESC, Pendleton[10], Learner-led[11], SHARP[12]	A short didactic on fundamentals of feedback and introduction and critique of some common feedback models.
		Simulated teaching activity: Participants watch a video of a teacher taking a student through basic suturing. Next the groups role-play using one of the feedback models. At each table, the 3 participants take the roles of teacher, student and observer; to role-play terminal feedback on the videoed task, followed by table debrief and whole class discussion.
Design and implantation of learning plan	Learning plan for skills teaching using task trainer	Individual written work using a learning plan template, followed by peer discussion and table discussion. Participants are then re-grouped according to their planned skills teaching and each group chooses one member's learning plan for implementation.
		Learning plans are role-played with recommended timing and participants take on roles as teacher, student and observer. Each group holds facilitated debriefs, followed by large class discussion.
Closing	Reflections and action plan	Facilitator-led group sharing on learning from the day, followed by individual written action plans.

POST-COURSE ASSESSMENT/ PROGRAM EVALUATION

Participants complete a feedback form at the end of the workshop where they are asked to rate the degree to which they met learning objectives, faculty effectiveness and workshop experience. Two months after the workshop, another email survey is sent asking them about their recall of the course learning objectives and implementation of personal action plans.

An interim qualitative program evaluation study was carried out after the first 4 workshops that forms the basis for this article. Study findings and all participant feedback have been used for iterative program improvement.

REFERENCES

1. Cheng A, Kessler D, Mackinnon R, et al. Reporting guidelines for health care simulation research: extensions to the CONSORT and STROBE statements. Adv Simul (Lond). 2016;1:25.
2. Cranton P, King KP. Transformative Learning as a Professional Development Goal. New Dir Adult Cont Educ. 2003:31-38.
3. Van Schalkwyk SC, Hafler J, Brewer TF, et al. Transformative learning as pedagogy for the health professions: a scoping review. Med Educ. 2019;53:547-58.

4. Mezirow J, Taylor EW. Transformative Learning in Practice: Insights from Community, Workplace, and Higher Education. Wiley; 2009.
5. Fitts PM, Posner MI. Human performance. Human performance. Oxford, England: Brooks/Cole; 1967.
6. Kolb DA. Experiential Learning: Experience as the Source of Learning and Development. Pearson Education; 2014.
7. Walker M PJ. Teaching in the theatre. . In: JWR P, editor. Teaching and learning in medical practice. Rickmansworth: Manticore Publishers:171-80.
8. McGaghie WC. Mastery learning: it is time for medical education to join the 21st century. Acad Med. 2015;90:1438-41.
9. Sawyer T, White M, Zaveri P, et al. Learn, see, practice, prove, do, maintain: an evidence-based pedagogical framework for procedural skill training in medicine. Acad Med. 2015;90:1025-33.
10. Pendleton D, & Hasler, J. Professional Development in General Practice. New York: : Oxford University Press Inc.; 1997.
11. Kneebone R, Nestel D. Learning clinical skills - the place of simulation and feedback. Clin Teach. 2005;2:86-90.
12. The London Handbook for Debriefing: Enhancing Performance Debriefing in Clinical and Simulated Settings.Imperial College, London. 2012
