# Supplementary material for: ‘It’s going to change the way we train’: Qualitative evaluation of a transformative faculty development workshop
Source: Perspect Med Educ. 2021 Oct 25;11(2):86–92. doi: 10.1007/s40037-021-00687-4 (PMC8543424; doi:10.1007/s40037-021-00687-4)
Supplement: Supplementary file 3 — Appendix 3: Topic guide [file 40037_2021_687_MOESM3_ESM.pdf]

Appendix 3: Topic guide

1. What do you remember about the OLTT workshop that you attended on (4 Nov 2017/20 Sep 2018/ 22 Oct 2019)?
    a. Why do you find this/these aspect(s) of OLTT memorable?
    b. Were any of this/these aspect(s) new to you?
    c. What did you like about this/these new aspect(s)?
    d. What did you not like about it/them?
2. We recognise that, in the classroom, you're usually the teacher. So, as a learner in the OLTT workshop, we're wondering if you spotted any of the types of activities OLTT facilitators used to engage the participants?
    i. Have you used any of these activities in your own teaching?
    ii. Could you elaborate?
3. Since attending OLTT, what do you think of your previous teaching practices?
4. Since attending OLTT, have there been any changes in the way you teach or design teaching programs? (trying out new roles)
    a. What's easy about maintaining these changes?
    b. What's hard about maintaining these changes?
    c. Has your teaching improved?
    d. How do you know?
    e. Sometimes teaching can improve in some regards but also worsen in other regards. Has your teaching worsened?
    f. How do you know?
5. *OLTT was a one-day workshop. What else would you like to know to advance your teaching skills?
6. Since attending OLTT have you attended other faculty development programs?
7. ***How much time do you spend on education?
    a. Do you see yourself as an educator?
8. How often do your colleagues talk to you about education matters?
    a. Who are they?
    b. Do you meet face-to-face, via email or …
    c. How does your interaction with other educators influence your teaching?
9. *Was the deck of slides about OLTT useful in helping you to recall the workshop?
    a. Could you elaborate?
10. **Do you have the habit of reflecting on your educational practices?
    a. Was this interview helpful in prompting you to reflect on your educational practices?

    b. Could you elaborate?

11. *You are the ___ person we have interviewed. And our study uses a theory to help us make sense of our data. One of the key concepts of that theory is that something of great impact shakes up someone's views of teaching – what's called a disorienting dilemma. Our findings so far indicate that most of our interviewees changed their educational practice gradually, and not because of a sudden event of great impact. What do you think about this in relation to your own experience? That is, would you support or challenge the concept of disorienting dilemma based on your experience of OLTT's impact on your educational practice
12. I might have overlooked to ask questions you think are important to the evaluation of OLTT's impact. Is there anything else you'd like to add?

The topic guide was amended 3 times during the study to elucidate codes and candidate themes: Changes for interview with:

* P6 – to include questions 5, 9, 11, 1.; Question 2 was expanded with 2(i) & (ii) to explore workshop impact and prior frames of reference. Question 4 was expanded with 4(a) & (b) to explore enactment of transformative learning.

**P7 to P10 to include question 10 about prior habits of reflection on educational practices

*** P11 to P17 to include question 7, exploring educator identity
