# Supplementary material for: ‘It’s going to change the way we train’: Qualitative evaluation of a transformative faculty development workshop
Source: Perspect Med Educ. 2021 Oct 25;11(2):86–92. doi: 10.1007/s40037-021-00687-4 (PMC8543424; doi:10.1007/s40037-021-00687-4)
Supplement: Supplementary file 4 — Appendix 4: Aide- Memoire: Powerpoint slide summary of key workshop topics and photographs to trigger participant recall during the interview [file 40037_2021_687_MOESM4_ESM.pdf]

SINGHEALTH · DUKE-NUS SCIENTIFIC CONGRESS

21 - 22 September 2018 Academia, Singapore

Academic Medicine: Translating Research into Clinical Practice

Pre-Congress Workshop

OPTIMISING LEARNING WITH TASK TRAINERS (OLTT)

20 Sep 2018 (Thu) | Academia, Level 2, PSL 2 & Surgical Simulation Lab | 8.30am - 3.00pm

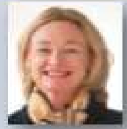

SPEAKER

Prof Debra Nestel

Professor of Surgical Education,
Department of Surgery
University of Melbourne, Australia

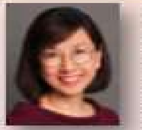

SPEAKER

Adj Assoc Prof Caroline Ong

Vice Chair (Postgraduate Education)
SingHealth Duke-NUS Surgery Academic Clinical Programme

INTRODUCTION

Teaching and learning of procedures and/ or surgical operations involves development of knowledge, psychomotor and cognitive skills. Simulation improves patient safety as it allows learning and practice away from the patient, so task trainers are often used to aid skills development. Task trainers may be used to teach basic skills (e.g. suturing sets, intubation models, catheter insertion models) or more advanced surgical skills (e.g. laparoscopic box trainers, computerised endoscopy units, animal and/or cadaveric models). Optimal use of task trainers in skills development involves using effective teaching methods in a well-designed curriculum that is integrated with the overall curriculum for the learners.

LEARNING OBJECTIVES

At the end of the workshop, participants will be able to :

- Discuss benefits of using task trainers in skills development
- Discuss limitations of using task trainers in skills development
- Describe models of skills development and training
- Describe the role of feedback in task trainer-based skills development
- Design a learning plan that uses a task trainer to support the development of a skill

WHO SHOULD ATTEND

- Faculty who are responsible for creating programs for development of procedural or surgical skills in their learners.
- All faculty who teach procedural and/or surgical skills using task trainers.

REGISTRATION FEES

$ 350.00 (Before GST)
Fee after subsidy: $ 250.00 (Before GST) - $100 is sponsored by Surgery ACP)

REGISTRATION

SingHealth PECT Faculty **will be funded** by the PECT Faculty Development Fund respectively.

- For Medical PECT Faculty, please indicate your interest through your respective ACPs.
- For SingHealth Nursing PECT Faculty, please indicate your interest through nursing@singhealthacademy.edu.sg.
- For SingHealth Allied Health PECT Faculty, please indicate your interest through alliedhealth@singhealthacademy.edu.sg.

The final application will be done through the Education Grant Call Application in May 2018.
For all others, please email the attached registration form to: sims@singhealth.com.sg

REGISTER BY 31 JUL 2018

ENQUIRIES

sims@singhealth.com.sg
DID: 6576 7102

Organiser: SingHealth DukeNUS ACADEMIC MEDICAL CENTRE

Secretariat: SingHealth Academy

Workshop Organisers: SingHealth DukeNUS Academic Medical Centre Institute of Medical Simulation; SingHealth DukeNUS Academic Medical Centre SURGERY

SingHealth DukeNUS ACADEMIC MEDICAL CENTRE

SingHealth DukeNUS ACADEMIC MEDICAL CENTRE | Institute of Medical Simulation

Selection of slides from workshop 2018

Optimizing learning with task trainers

SIMS 20 Sep 2018
Singhealth-Duke NUS Scientific Congress

Invited faculty

Debra Nestel

PhD FoME FSSH

Professor of Simulation Education in Healthcare
Monash University
Professor of Surgical Education
University of Melbourne

Local faculty

Caroline Ong

MBBS FRCS M Surgical Ed

VC Education (Postgrad) Surgery ACP
Senior Consultant Paediatric Surgery KKH

Chong Shin Yuet

MBBS (Hons) M Med (Anaes)

Director Simulation Training Anaesthesiology Periop Svc ACP
Senior Consultant Anaesthesia SGH /SKH

Sharon Ong

MBBS M Med (Anaes)

APD Anaesthesia Residency
Senior Consultant, Intensive Care & Anesthesia SGH/ SKH

Aim

- To explore educational processes associated with task trainers and skills development using simulation

SingHealth DukeNUS
ACADEMIC MEDICAL CENTRE

Learning objectives

After this workshop, you will be able to:

1. Describe models of skills development and training
2. Discuss benefits of using task trainers in skills development
3. Discuss limitations of using task trainers in skills development
4. Describe the role of feedback in task trainer-based skills development
5. Design a learning plan that uses a task trainer to support the development of a skill

Sessions

1. Simulation fundamentals
2. Overview of skills development
3. Role of feedback in learning
4. Design and implement a lesson plan
5. Reflections, closing and action plan

Simulation fundamentals

Session 1

Simulation phases

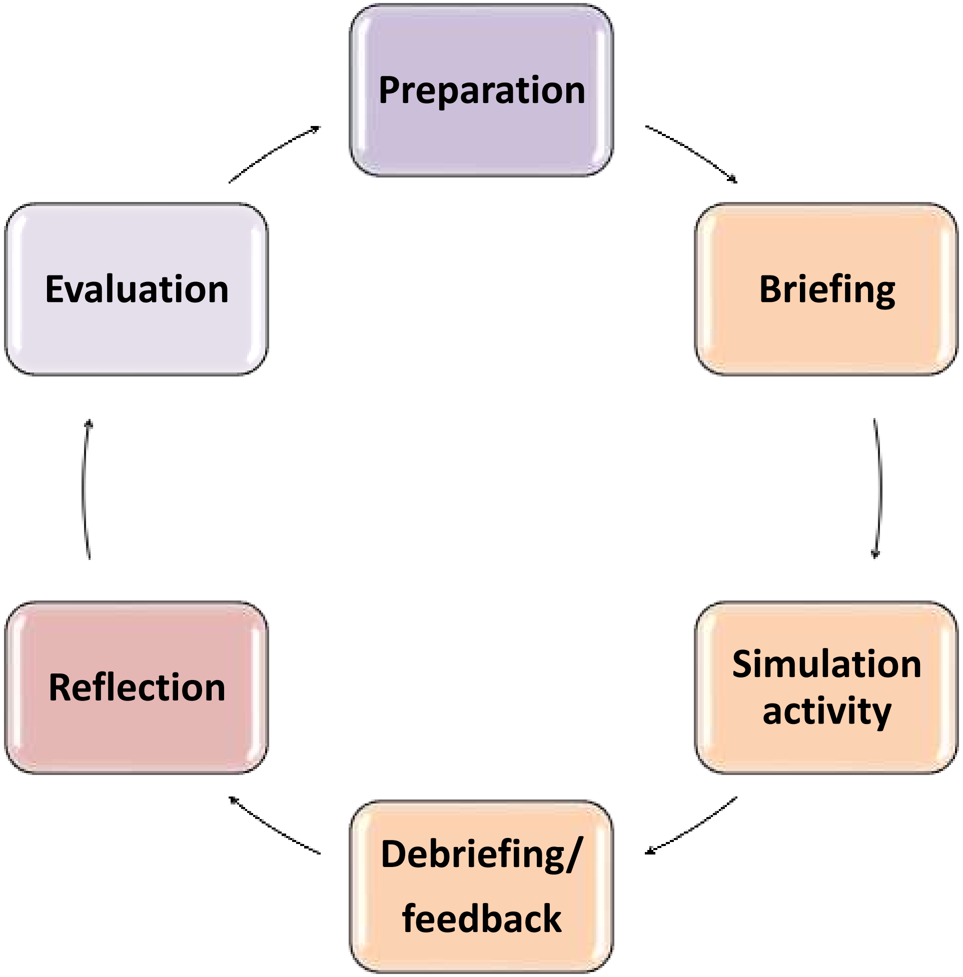

www.nhet-sim.edu.au

Pre-reading

Wulf, G., et al. (2010).

Motor skill learning and performance: a review of influential factors.

Medical Education, 44(1), 75-84.

Hatala, R., et al (2013).

Feedback for simulation-based procedural skills training: a meta-analysis and critical narrative synthesis.

Advances in Health Sciences Education, 1-22.

Nicholls, D., et al. (2016)

Teaching psychomotor skills in the twenty-first century: Revisiting and reviewing instructional approaches through the lens of contemporary literature.

Med Teach, 38(10), 1056-1063.

SingHealth DukeNUS ACADEMIC MEDICAL CENTRE

Task trainers

- Task trainers focus on supporting learning of a particular task or motor skill

- “Skills such as picking up objects, suturing, knot tying, applying clips, inserting an endotracheal tube and applying a tourniquet are examples of such tasks.”

(Kunkler, 2006)

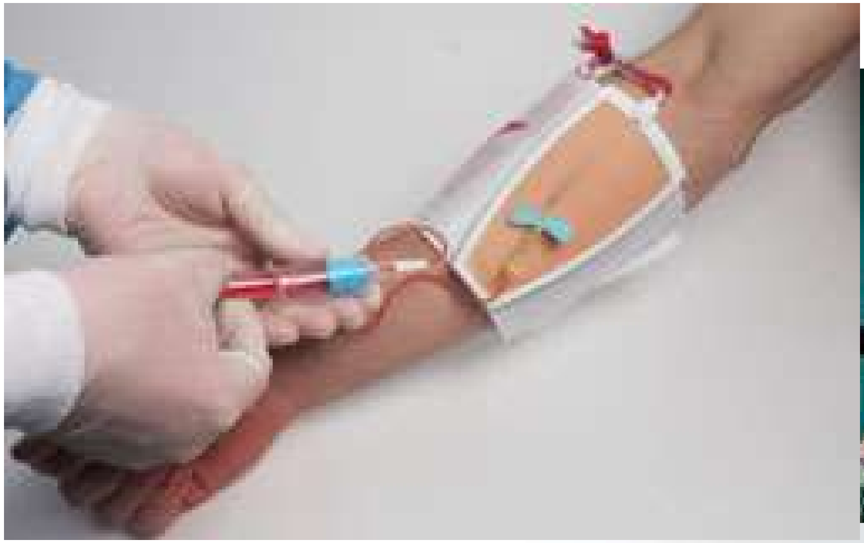

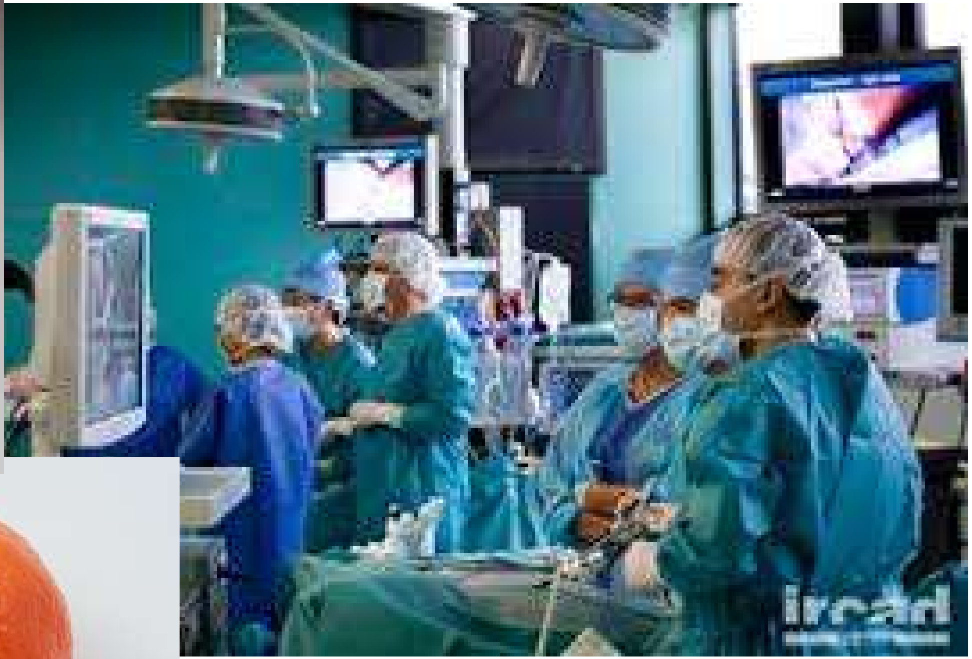
ircad

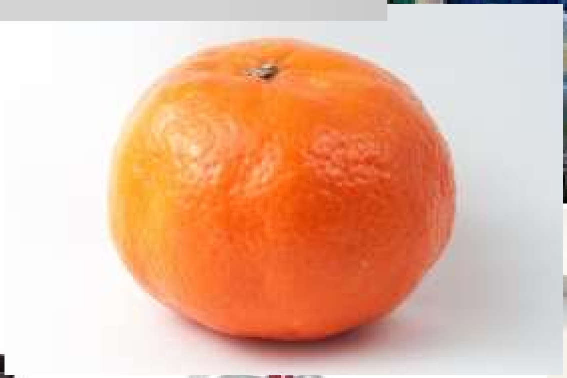

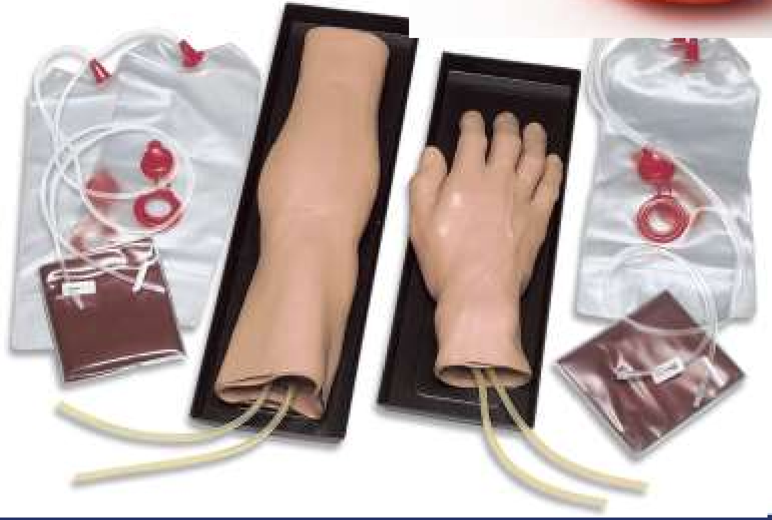

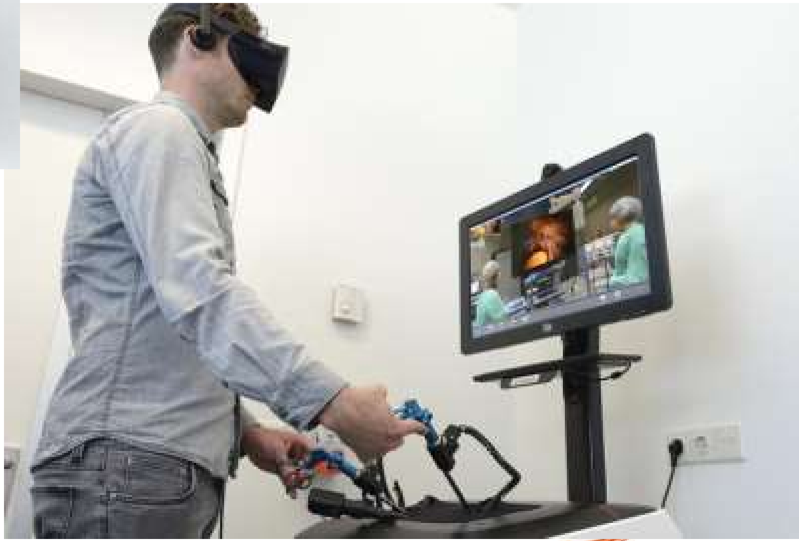

Activity #3 and #4: Task trainers

- Which task trainers do you use?
- Select one of the task trainers you use to answer the next questions:
- What are the benefits?
- What are the limitations?

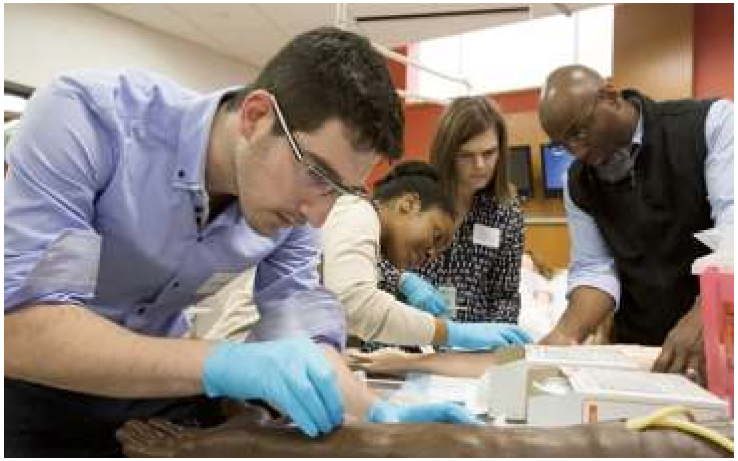

Benefits / Limitations

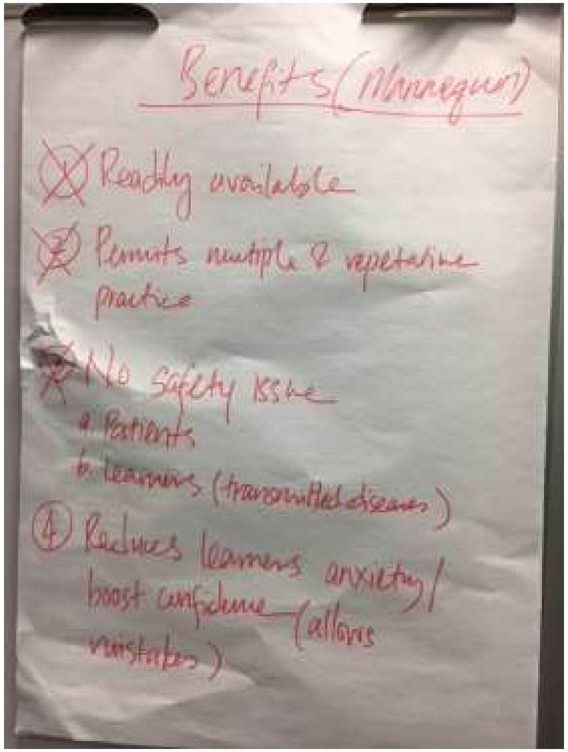

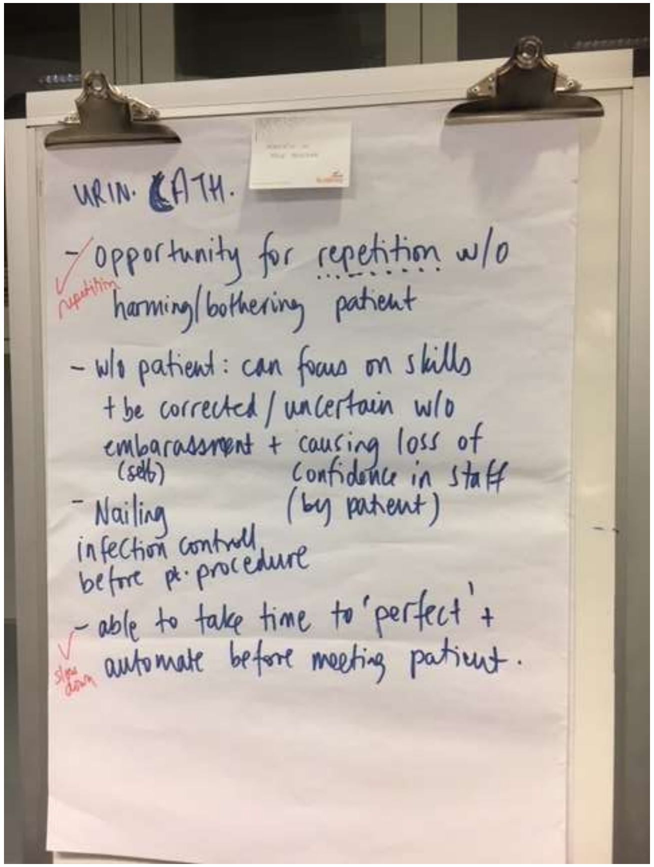

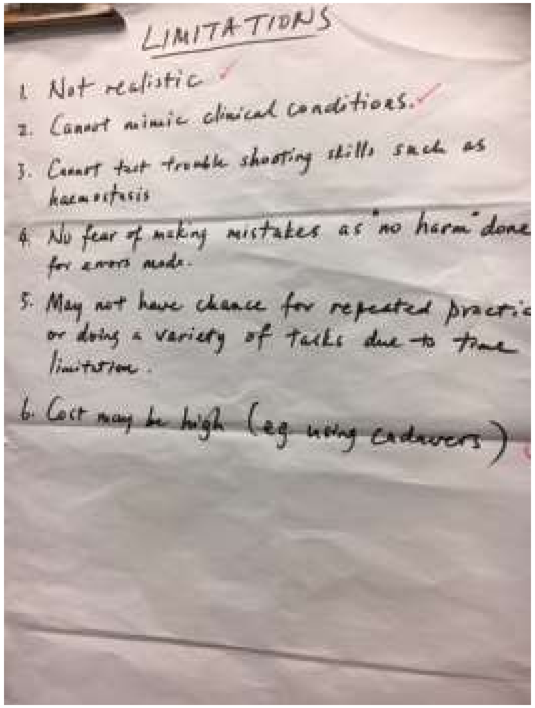

Overview of skills development

Session 2

Fitts (1964); Fitts and Posner (1967)

Stages of learning	Characteristics	Attentional demands
Cognitive (verbal)	Movements are slow, inconsistent and inefficient<br>Considerable cognitive activity required	Large parts of the movement are consciously controlled
Associative	Movements are more fluid, reliable and inefficient<br>Less cognitive activity is required	Some movements are controlled consciously and some automatically
Autonomous (motor)	Movements are accurate, reliable and efficient<br>Less or no cognitive activity is required	Movement is largely controlled automatically

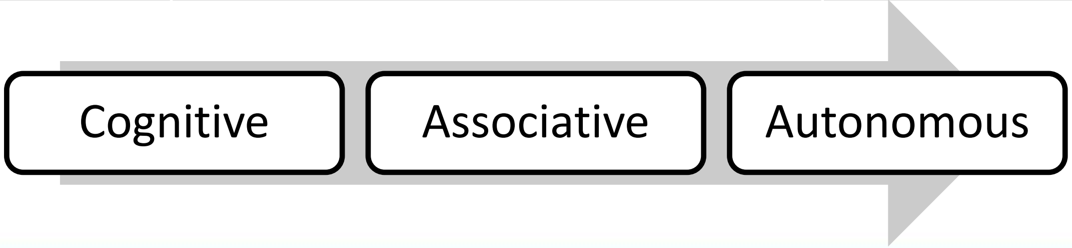

Kolb (1984)

Experiential learning cycle

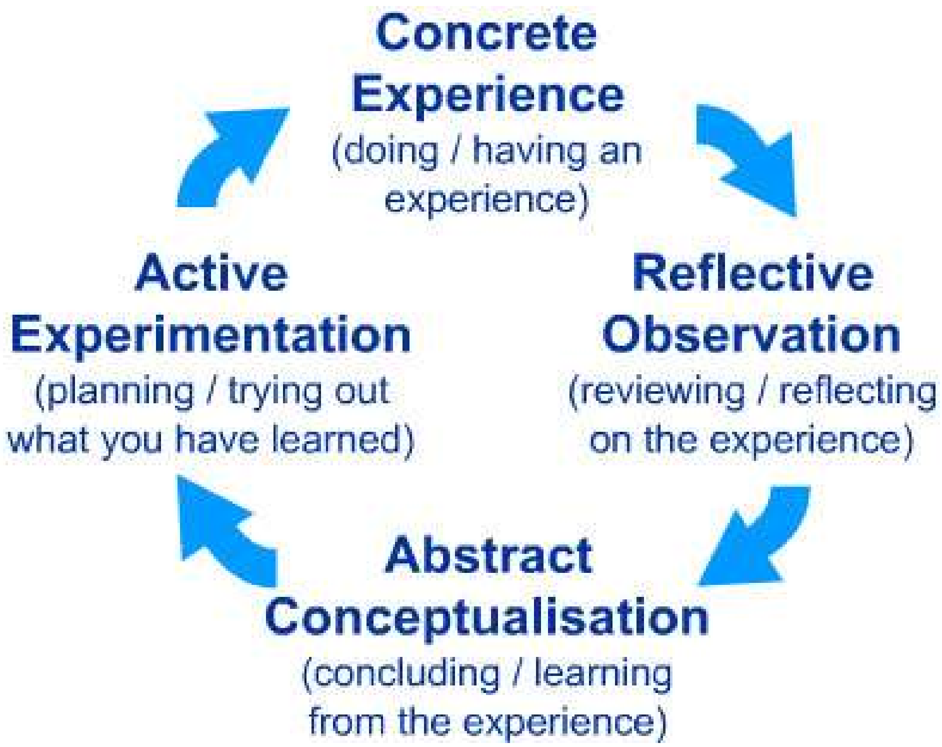

from https://www.simplypsychology.org/learning-kolb.html

Activities:

Writing in course workbook and group discussions

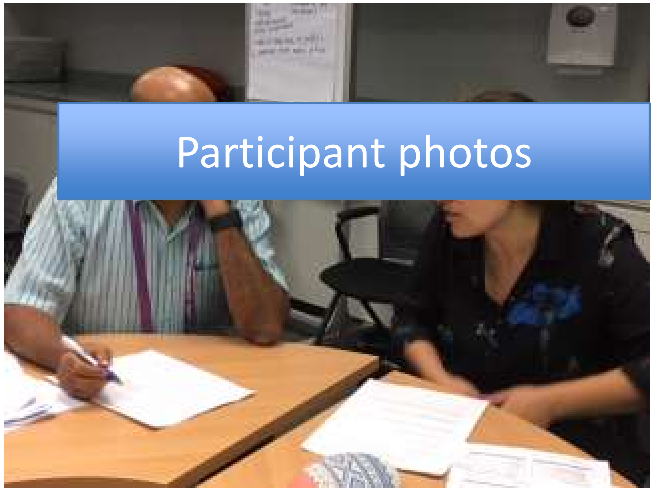

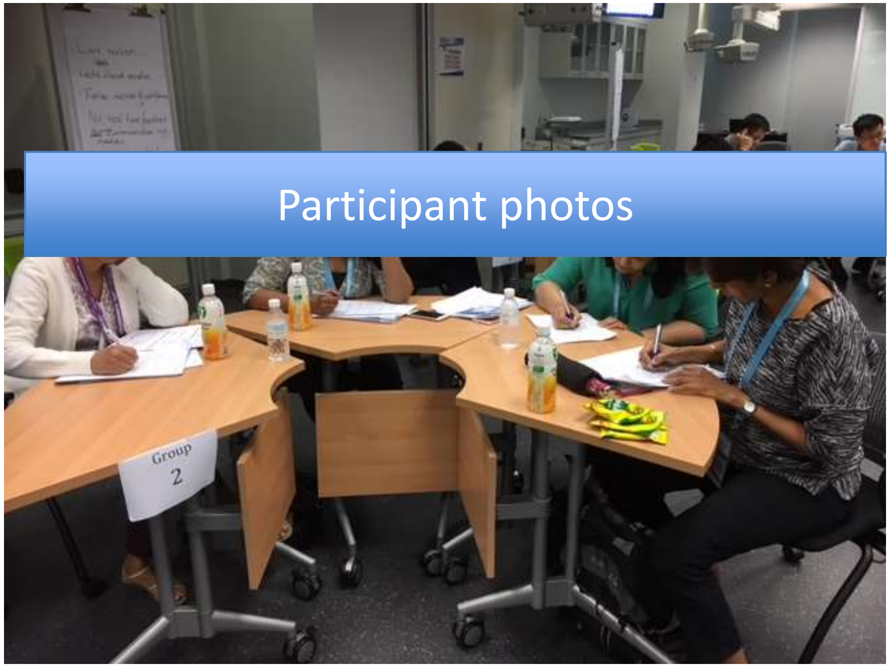

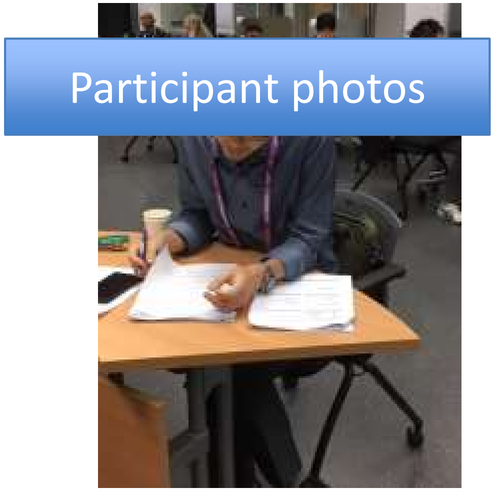

Peyton (1998)

4 stage approach to skills development

1: Surgeon demonstrates at normal speed → 2: Surgeon performs and explains → 3: Surgeon performs, learner explains → 4: Learner performs and explains to surgeon

SingHealth DukeNUS ACADEMIC MEDICAL CENTRE

Activity #7

- Groups of 3
- Teach the student to suture/venepuncture using Peyton
- 10 minutes only

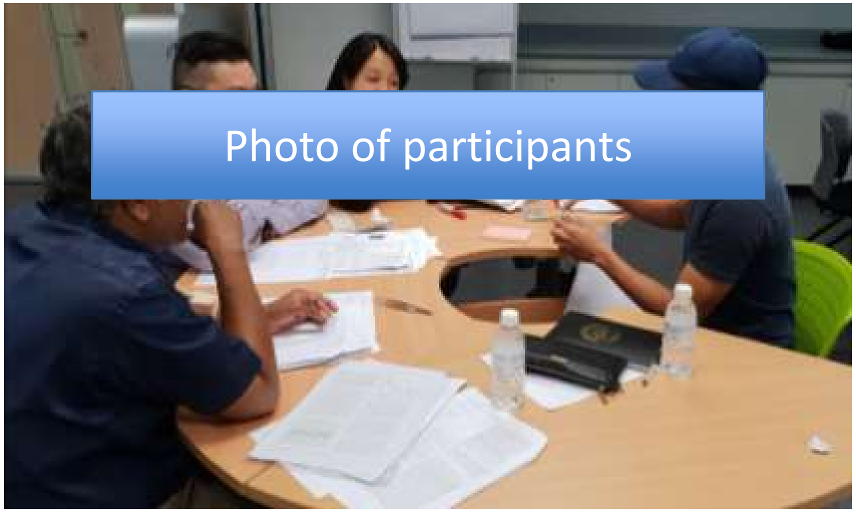

SingHealth DukeNUS
ACADEMIC MEDICAL CENTRE

Simulation-based mastery learning

1. Baseline, or diagnostic testing
2. Clear learning objectives, sequenced
3. Engagement in educational activities
4. A set minimum passing standard (e.g., test score)
5. Formative testing to gauge unit completion at a preset minimum passing standard for mastery
6. Advancement to the next educational unit given measured achievement at or above the mastery standard
7. Continued practice or study on an educational unit until the mastery standard is reached

(McGaghie, 2015)

FOR LATER REFERENCE

SingHealth DukeNUS ACADEMIC MEDICAL CENTRE

Evidence-based framework

Pedagogical framework for procedural skill training

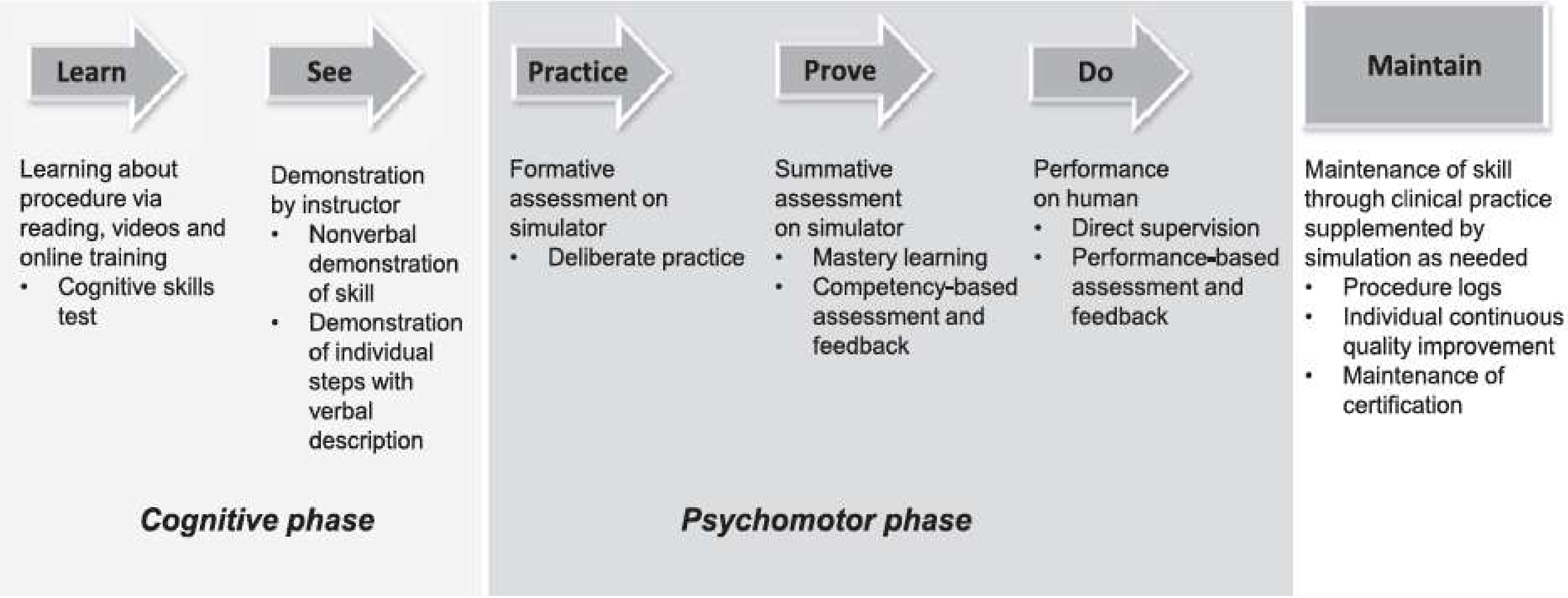

Sawyer et al, 2015

SingHealth DukeNUS ACADEMIC MEDICAL CENTRE

Activity + group photo

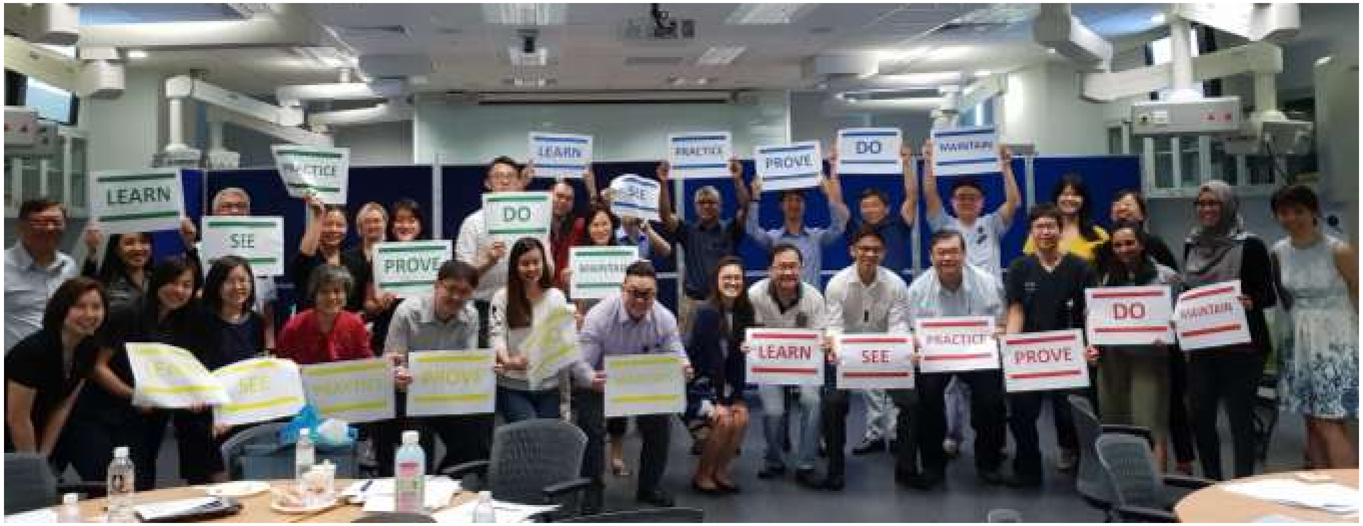

Evidence based framework for procedural skills training:
Sawyer et al 2015

Role of feedback in learning

Session 3

Models & approaches

1. DESC
2. Pendleton
3. Learner-led feedback
4. SHARP

Others…

Video

Surgery ACP Surgical Skills Videos

<u>Teaching Simple Suturing (Short Version - 4 min)</u>

- The learner is a final year medical student, who has attended 2 suturing workshops prior to this video.
- The teacher is a junior faculty who has been teaching basic surgical skills for four years.

Consent from volunteers have been obtained for this educational video
Property of Surgery Academic Clinical Programme 2016

SingHealth DukeNUS ACADEMIC MEDICAL CENTRE
SURGERY

Activity #8 : Video review

- Refer to the video
  - Aim of the simulation activity is to support the student in learning a skill
- Simulation
  - Groups of 3 – teacher, student, observer
  - 5 minutes of feedback as if you are the teacher and the student in the video
  - 10 minutes discussing approach used – what was helpful/areas for development

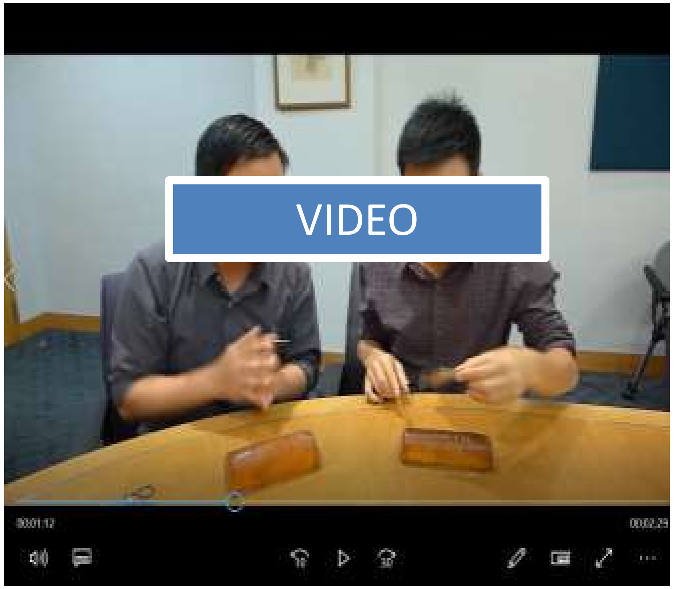

SingHealth DukeNUS
ACADEMIC MEDICAL CENTRE

Design and implement a lesson plan

Session 4

Writing learning objectives

Use behavioural terms

- Demonstrate
- Perform
- Apply
- Complete
- Achieve
- Master
- Refine
- Accomplish
- Show
- Arrange
- Handle
- Use

After the activity, learners will be able to:

- **Describe** the relevant anatomy for skin and tissues of the lower leg
- **Demonstrate** sensitive handling of tissue
- **Handle** instruments correctly

Writing lesson plan

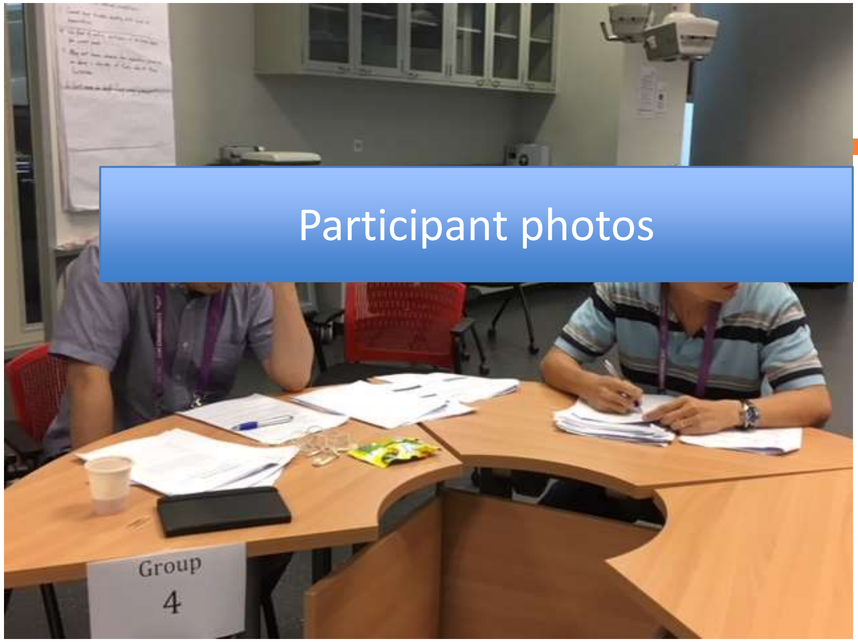

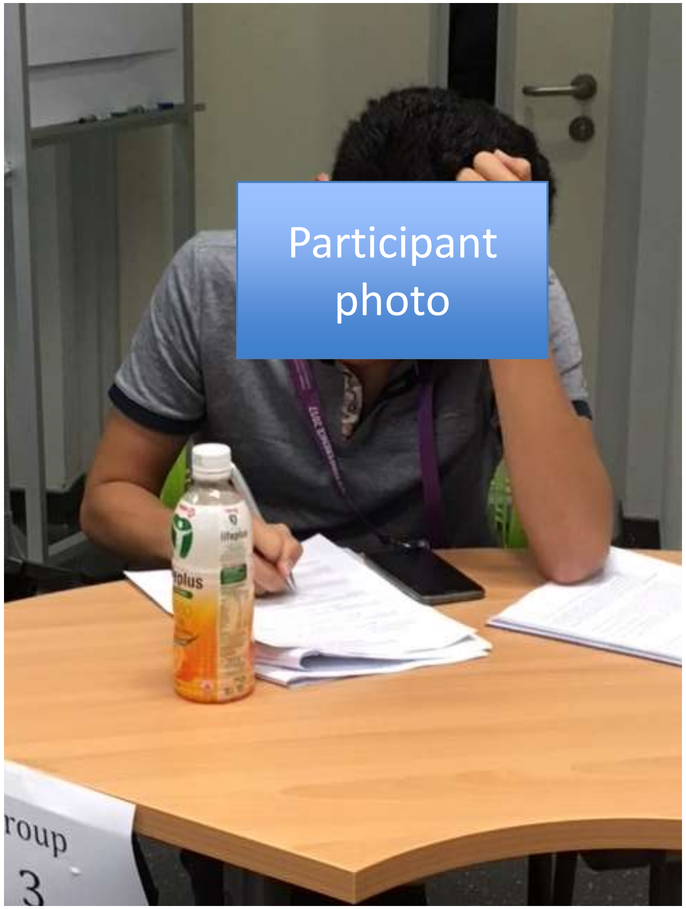

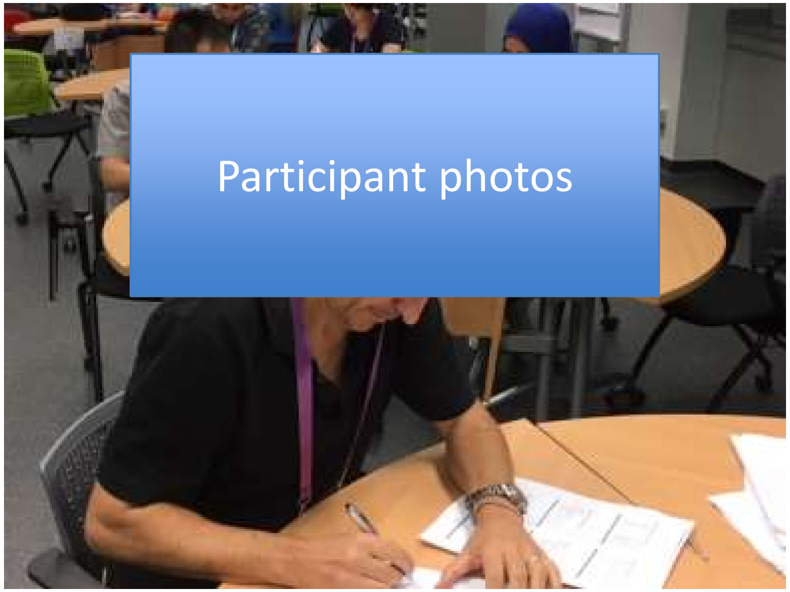

SingHealth DukeNUS
ACADEMIC MEDICAL CENTRE

Implement the lesson plan

- Preparation
- Briefing
- Simulation activity
- Feedback/debriefing
- Reflection (not possible in the workshop)
- Evaluation (not possible in the workshop)

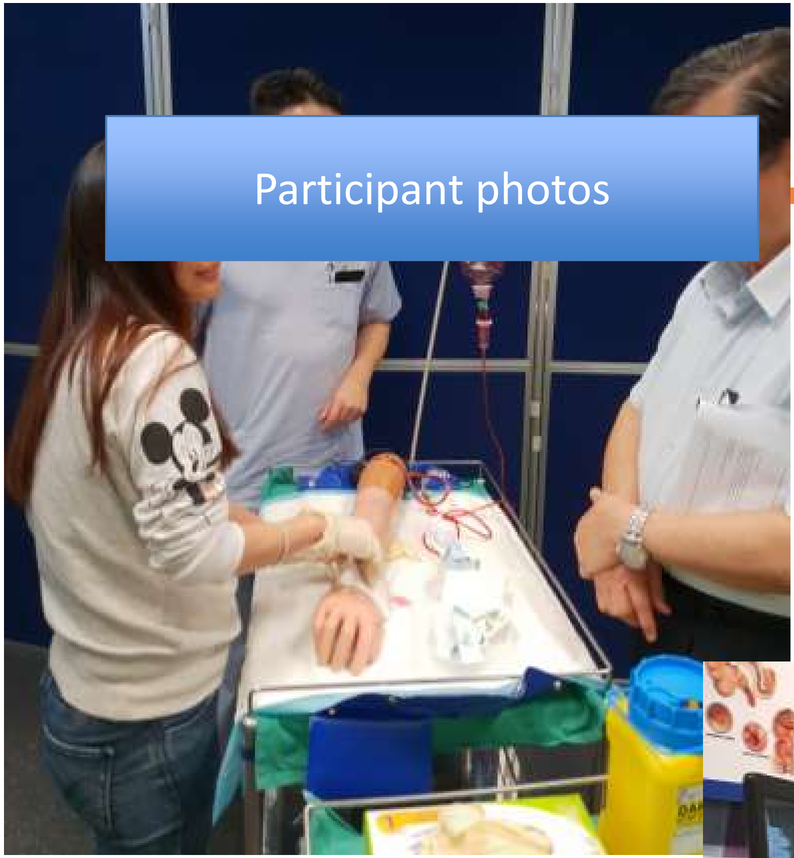

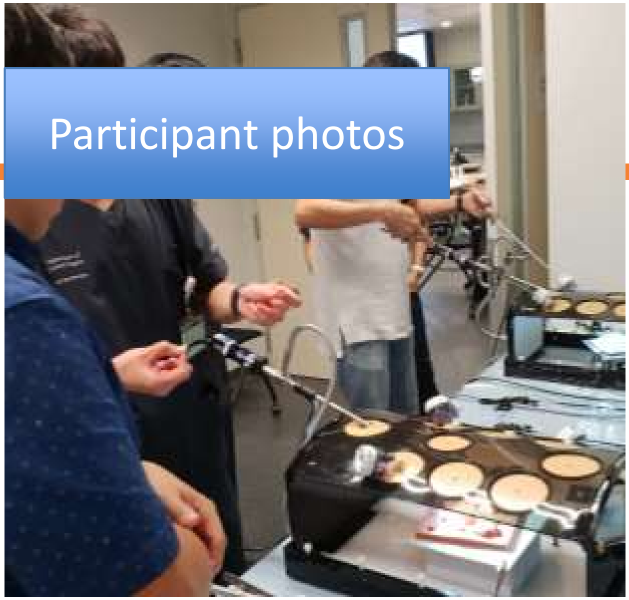

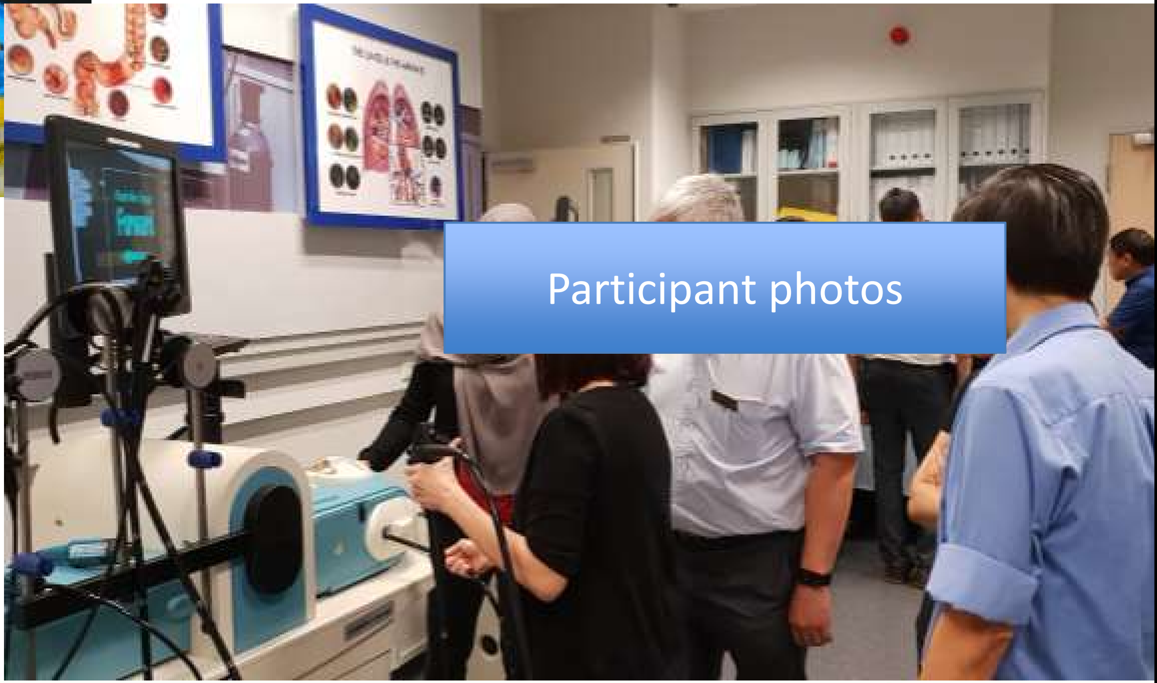

Implementing the lesson plan

Reflections, closing & action plan

Session 5
