# Supplementary material for: ‘It’s going to change the way we train’: Qualitative evaluation of a transformative faculty development workshop
Source: Perspect Med Educ. 2021 Oct 25;11(2):86–92. doi: 10.1007/s40037-021-00687-4 (PMC8543424; doi:10.1007/s40037-021-00687-4)
Supplement: Supplementary file 5 — Appendix 5: Summary table of study themes and subthemes with representative quotes [file 40037_2021_687_MOESM5_ESM.docx]

Appendix 5: Summary table of study themes and subthemes with representative quotes

| **THEME 1: Perspectival shift in educational practice**  A substantive shift in concepts of learning and/or teaching based on experiences in the workshop and subsequent application in practice. | |
| --- | --- |
| **1.1 New ideas, practices, or approaches** | |
| Idea(s) that profoundly changed the learner’s conceptualization of learning and/or teaching. | *1.1.1 My takeaway from the workshop was that I managed to learn a new skill which I had never done before, in half an hour [during a workshop activity]. It was impressive to me. So that method if applied for new [AHP] would be very effective. (P17, AHP)* |
| Resources and/or tools that help the learner to make concrete changes to teaching practice to align with new understandings. | *1.1.2[Learning plan template] was new...it's definitely useful because it methodically lists down what are the various components that you need to take into consideration when you are coming up with the lesson plan. ... it helps to crystallize and summarize what is out there so that we're not left to struggle and figure out... what works what doesn't work. (P16, doctor)* |
| **1.2 New insights into existing concepts about education** | |
| Workshop experience of taught educational concepts reinforced learning. | *1.2.1 I realize ‘hey education is more than sitting down and taking notes and trying to remember’ because that course help[ed] me realize that it is not the sitting down memorizing that helps the learning. It's actually the ‘doing and keep reinforcing over and over again’ that helps. (P13, AHP)* |
| Exposure to educational frameworks enhanced understanding and offered a rationale for teaching practice. | *1.2.2 Previously it's just like you're doing it without realizing that you're applying it. But now I manage to understand the definition of these steps and what it actually means. Because last time I would have probably done it without knowing... I think they covered quite [a lot] of theories which I wasn't aware of. (P17, AHP)* |
| **1.3 New** **relationships** **foster critical discourse for learning** | |
| Opportunities for new connections between participants improved learning through critical discourse. | *1.3 The people attending the course [were] from all different disciplines. So there were other more senior doctors from gastroenterology, anesthesia...surgeons from other institutions... coming together into different small groups and discussing... I felt quite useful. (P3, doctor)* |
| **THEME 2: Deepening, validating, and re-affirming current practices**  The workshop experience serves to clarify and structure prior ideas on teaching-learning and to affirm the associated beliefs and values. | |
| Repeat exposure through the workshop, of concepts previously formally learned or experienced, served to affirm beliefs and values about learning and/or teaching. | *2.1 Actually I was also telling my colleagues who attended the workshop together with me…this is actually what we did in the past, just not that systematically. (P11, doctor)* |
| Learner experiences in the workshop helped to clarify and structure the approach to learning and/or teaching. | *2.2 After the learning there's always the debrief, right, so it's during that debrief where you can kind of understand the view from the learner and how they felt during the whole training process. So it helps the teacher also. (P5, doctor)* |
| **THEME 3: Being and/or becoming an educator**  Health professionals (HPs) identify as clinicians and not all HPs have developed an educator identity in addition to the HP identity. Attunement and growth mindset is necessary for transformative learning. | |
| **3.1 Having or developing an educator identity in addition to the HP identity** | |
| HPs primarily identify professionally as doctor, nurse or AHP, even though teaching is part of the HP role. Only some HPs have developed an educator identity that is additional to the HP identity. | *3.1.1 I'm not really on the education pathway officially but this is just something I have been having an interest in … I always consider myself an amateur educator. (P11, doctor)* |
| Teaching ability is often conflated with clinical seniority | *3.1.2 I didn't join [institution] with a teaching background or anything like that…because I'm one of the seniors in the force and they expect me to… with the kind of experience that I've gathered… to convey that experience to the younger [AHPs]. (P8, AHP)* |
| Formal appointment and/or formal faculty development reassures HP educators about their teaching practice. | *3.1.3 It's like your boss just tells you to do this but if you can't, it becomes a bit terrifying to know that the training of your future juniors and all that is solely decided by one person who decided, ‘oh, it should be done this way’. Now at least after attending this, I have some backbone and I can say that this is designed and planned with some evidence in mind and proper framework. (P5, doctor)* |
| **3.2 Attunement and growth mindset are necessary for transformative learning** | |
| The passionate educator demonstrates regular self-reflection on teaching practice, seeks out opportunities to try new teaching-learning methods, solicits feedback and discusses education matters with others. | *3.2.1* *You pick up on the things you can improve on ... if you attend like a talk... as a bystander, you see how they teach in ‘that’s a good method’, then… I might use it in my own teaching in the future. (P3, doctor)*  *3.2.2 It's always useful to exchange notes and see what other hospitals are doing. And whether they have gone through something similar like [this workshop]… teaching is a very lonely journey if you are the only educator and nobody believes in it. (P5, doctor)* |
| **THEME 4: Valuing faculty development that accommodates HPs’ multiple communities of practice**  Faculty development for HPs should recognize competing clinical priorities and the workplace community of practice. | |
| **4.1 Design of faculty development sensitive to HPs’ complex clinical/workplace needs** | |
| Due to completing clinical priorities, HPs value focused, relevant formal faculty development programs with regular refreshers. | *4.1. It's easy to slip back [to old practices] because... clinical commitments are very many, so sometimes you don't get time…to plan… but since I realized … the importance of it, I do try to take time out as much as possible… I think we should have refreshers … regularly maybe once a year… or something like that. (P9, doctor)* |
| **4.2 The workplace educator community of practice influences transformative learning gained through formal faculty development** | |
| HPs engage on education matters opportunistically, with clinical colleagues that form their workplace community of practice. | *4.2.1 My own supervisors, nursing supervisors. Besides [Doctor A], other doctors as well. We always discuss [teaching]. (P2, Nurse)* |
| The educator expertise available in the workplace and the value placed on education affects the support given to HP educators and the critical discourse available. | *4.2.2 We actually do it [curriculum design planning] during the clinical time. So we have to ask our bosses whether can he or she let us go for that few hours so that we can meet up to discuss…So if I'm away, then the rest [of my colleagues] will have to probably scan a little bit more patients. (P14, AHP)*  *4.2.3 [Referring to her mentors] Like for example, handling difficult students...Like approaches to structuring a curriculum, I also learned a lot from them…because they are more experienced… what students need... what sort of classroom teaching, what sort of pattern of clinical teaching and even their assessment styles. (P12, doctor)* |
